# Supplementary figures and images for: A Population Genomics Approach to Assessing the Genetic Basis of Within-Host Microevolution Underlying Recurrent Cryptococcal Meningitis Infection
Source: G3 (Bethesda). 2017 Feb 10;7(4):1165–76. doi: 10.1534/g3.116.037499 (PMC5386865; doi:10.1534/g3.116.037499)

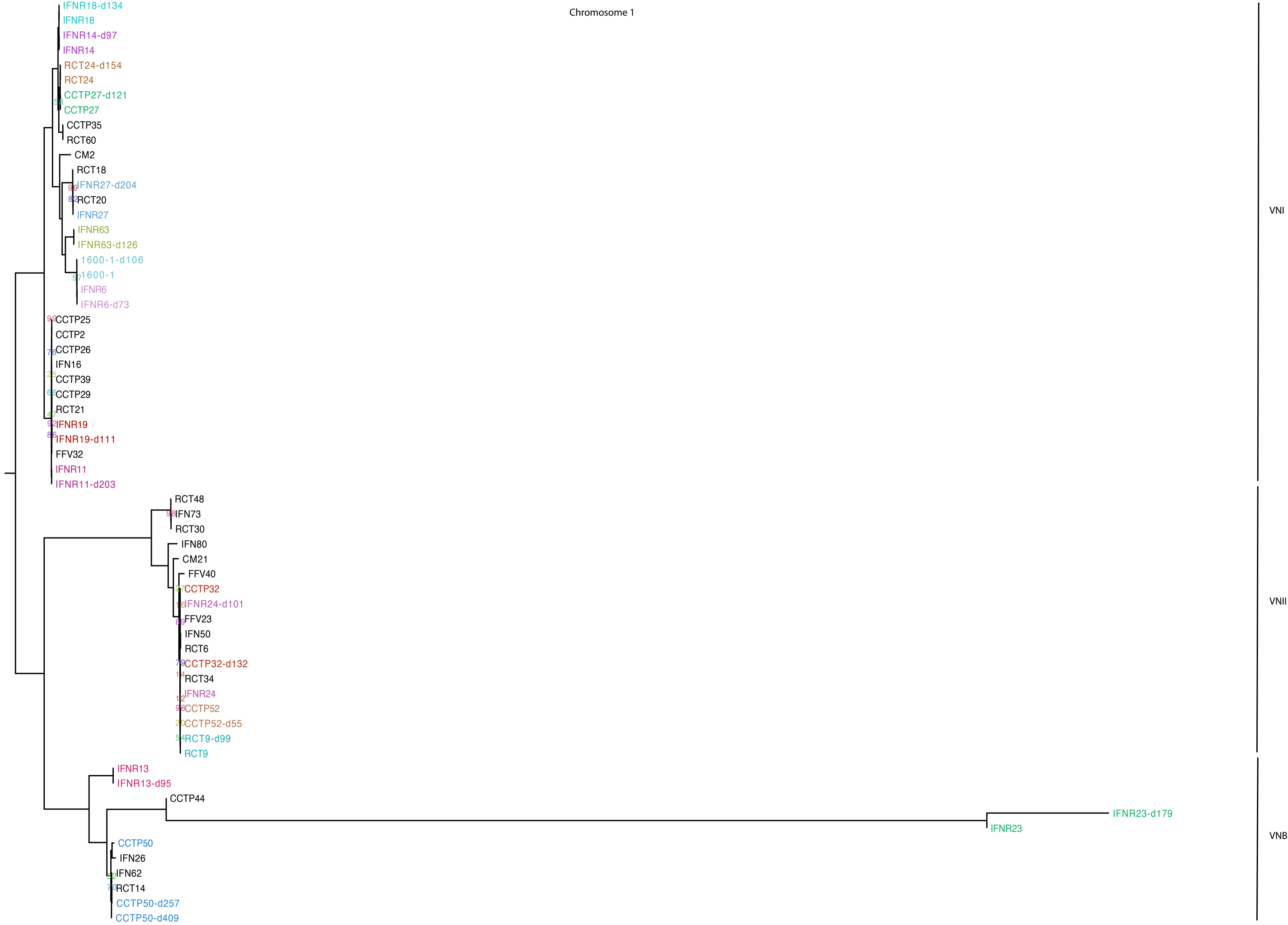

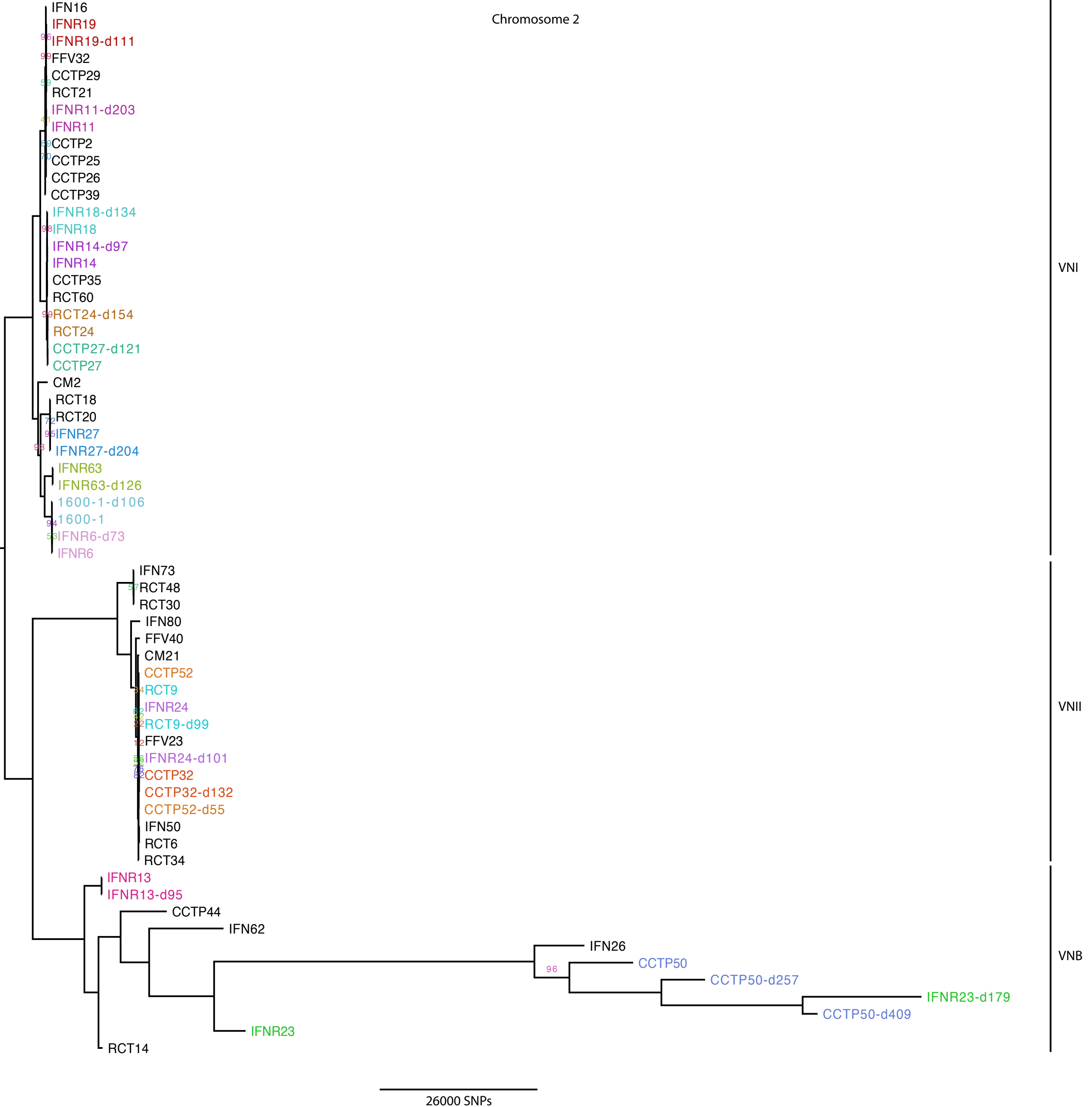

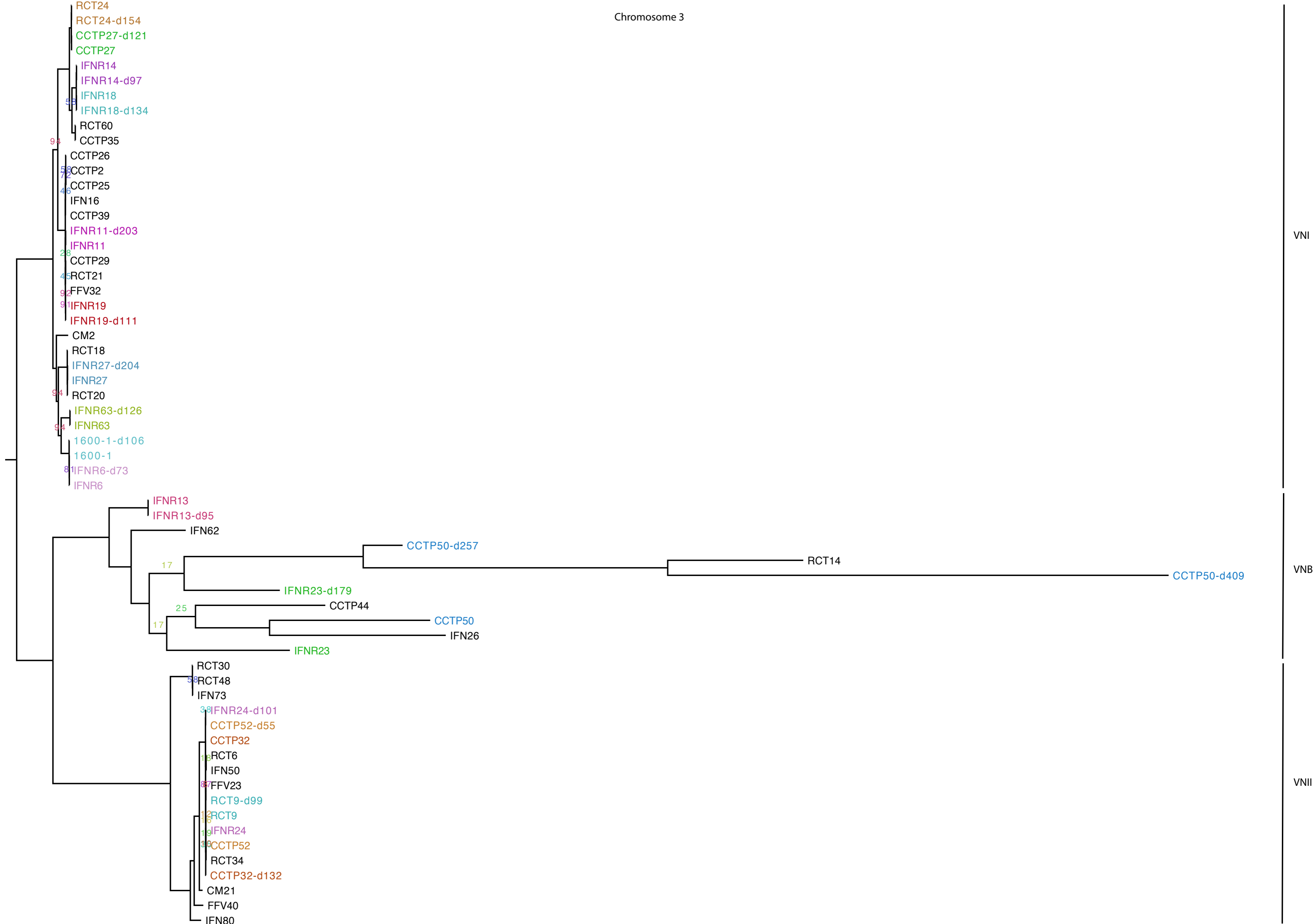

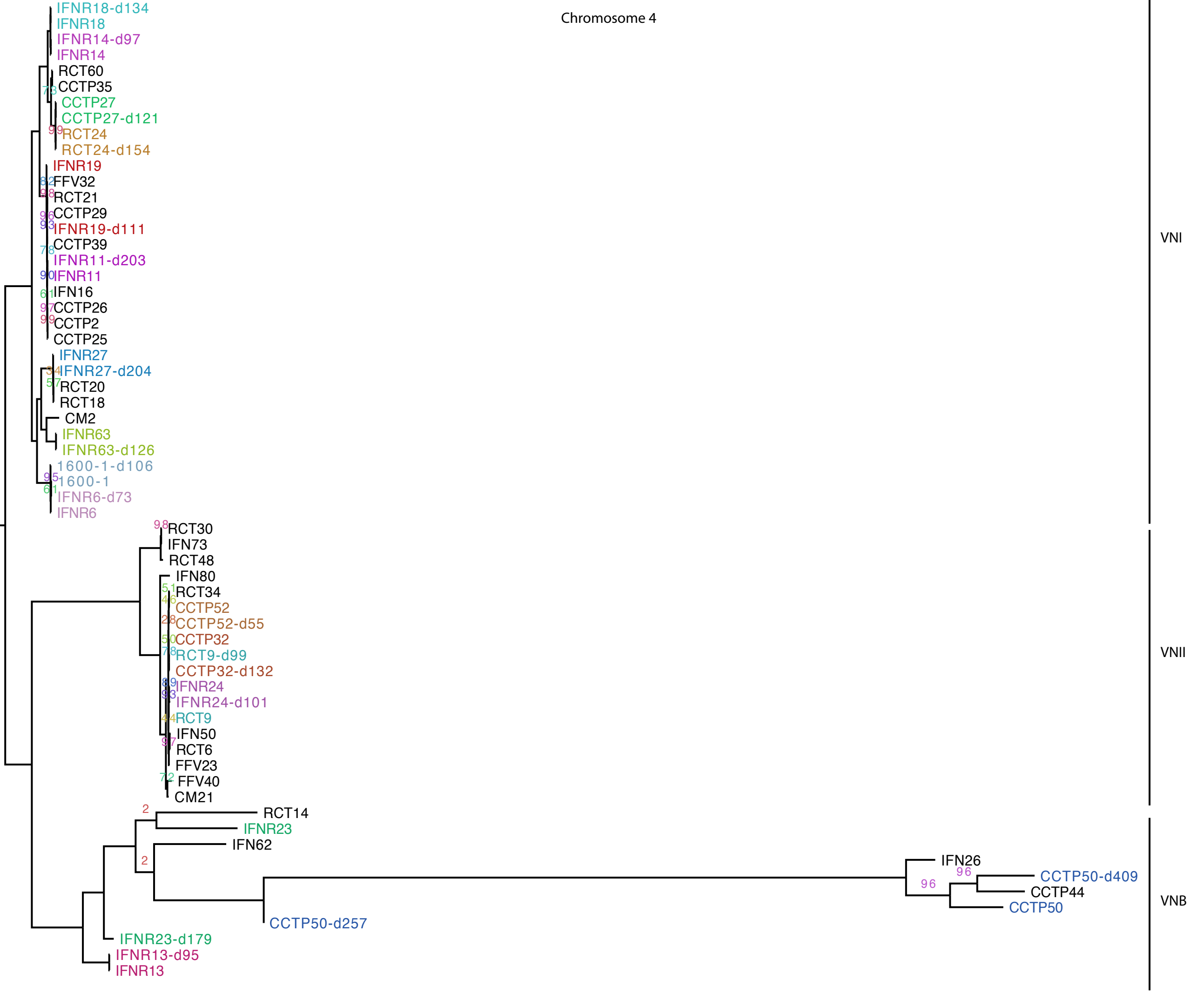

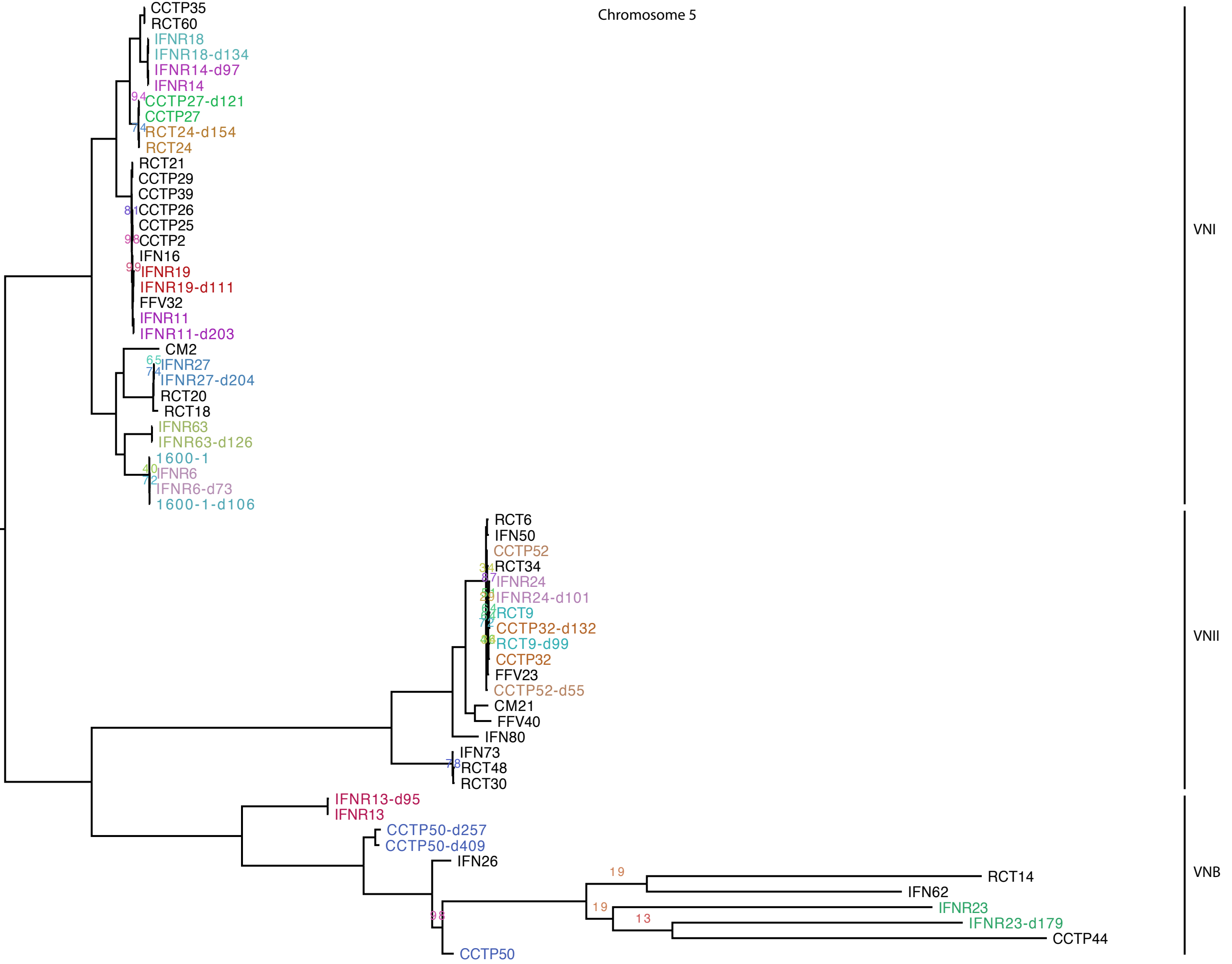

74500 SNPs

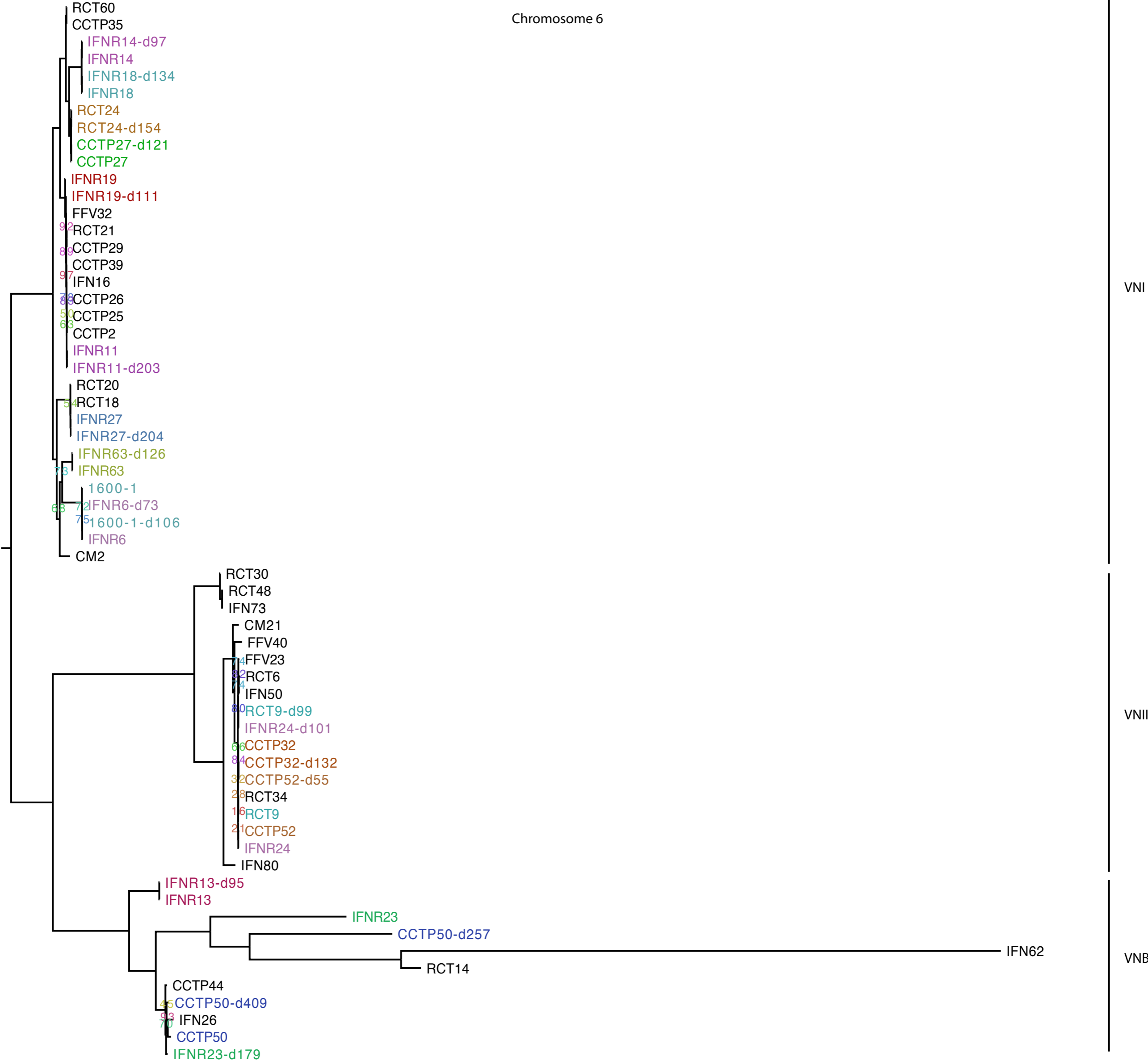

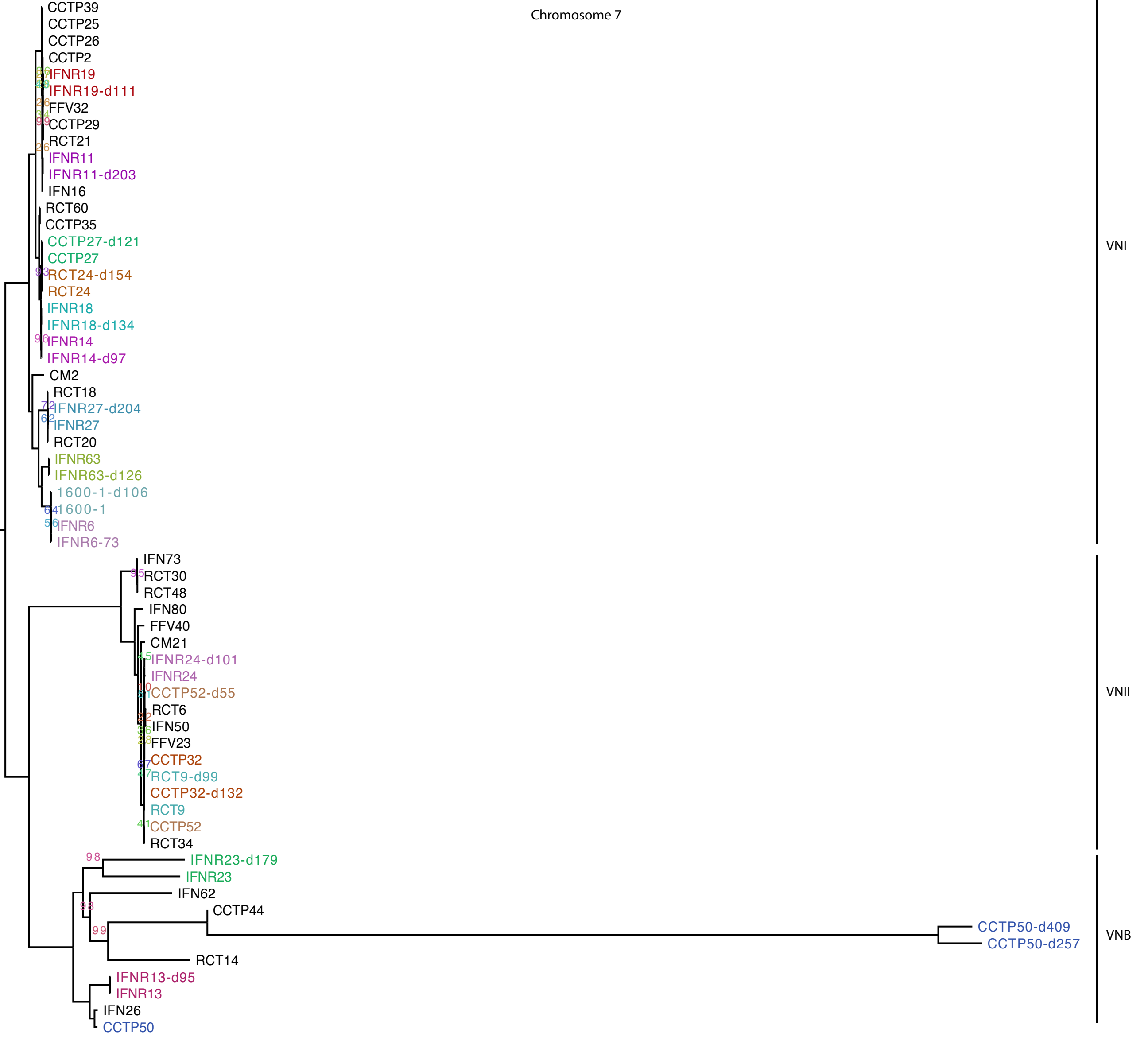

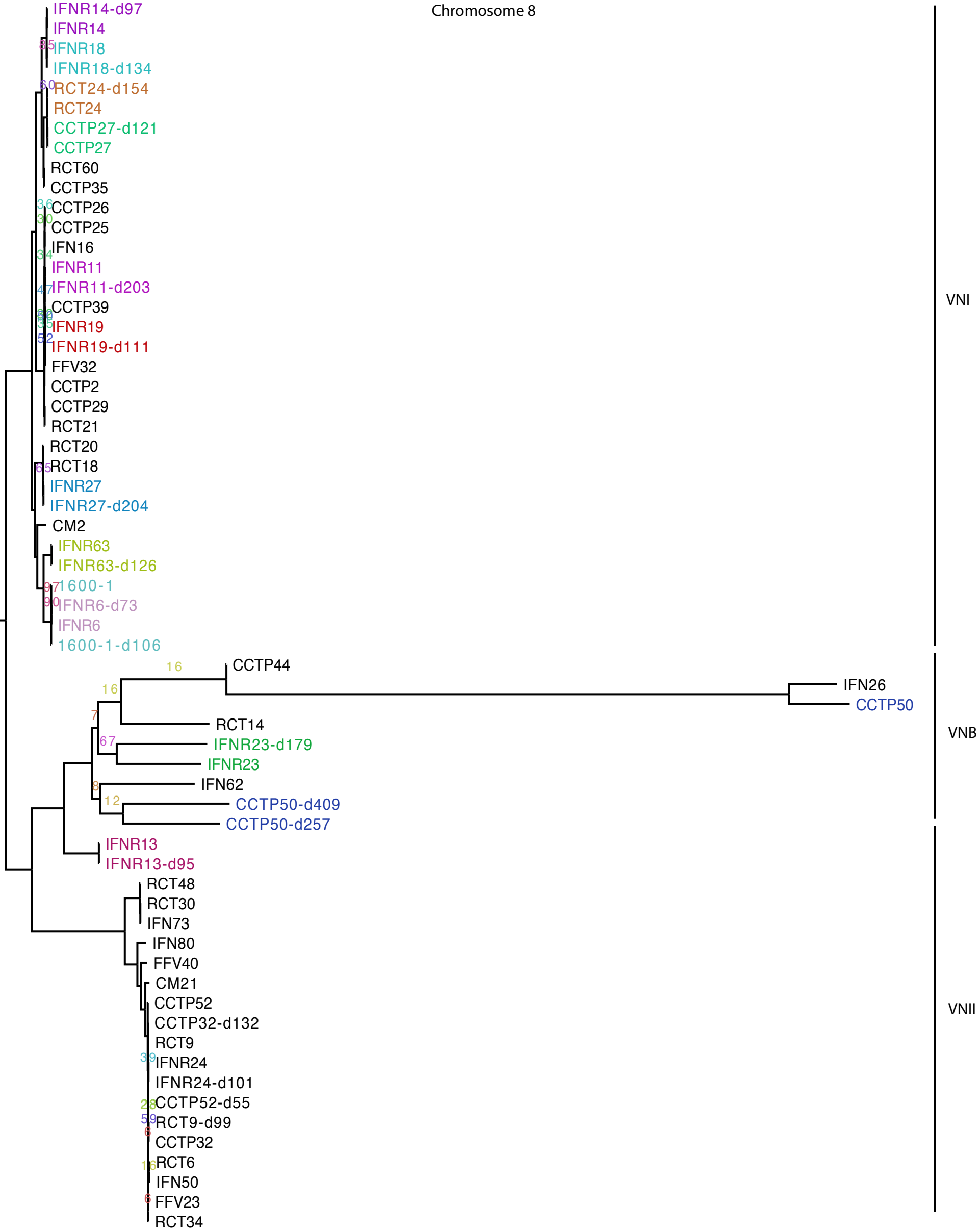

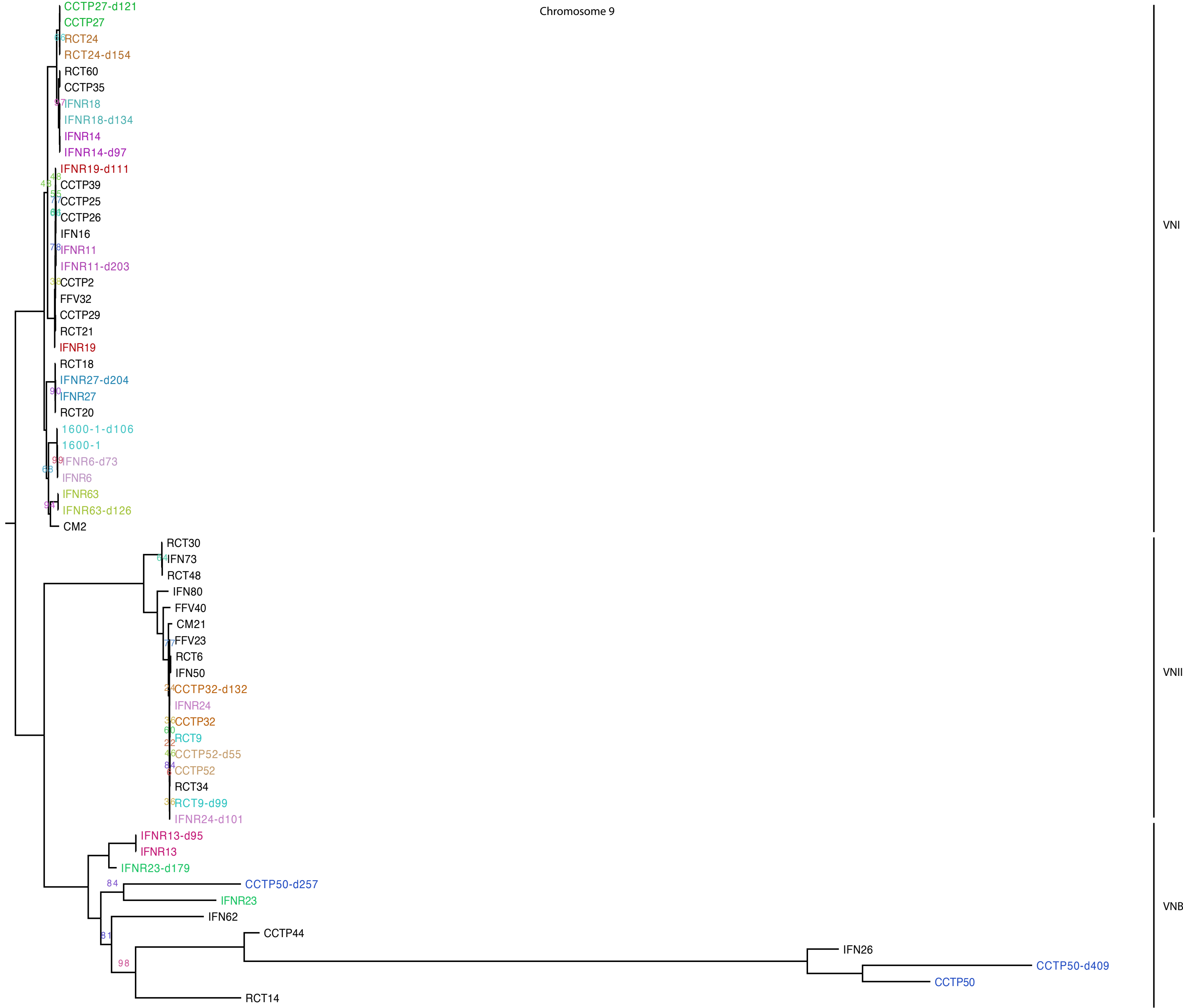

## Chromosome 10

VNI

VNE

VNI

18000 SNP

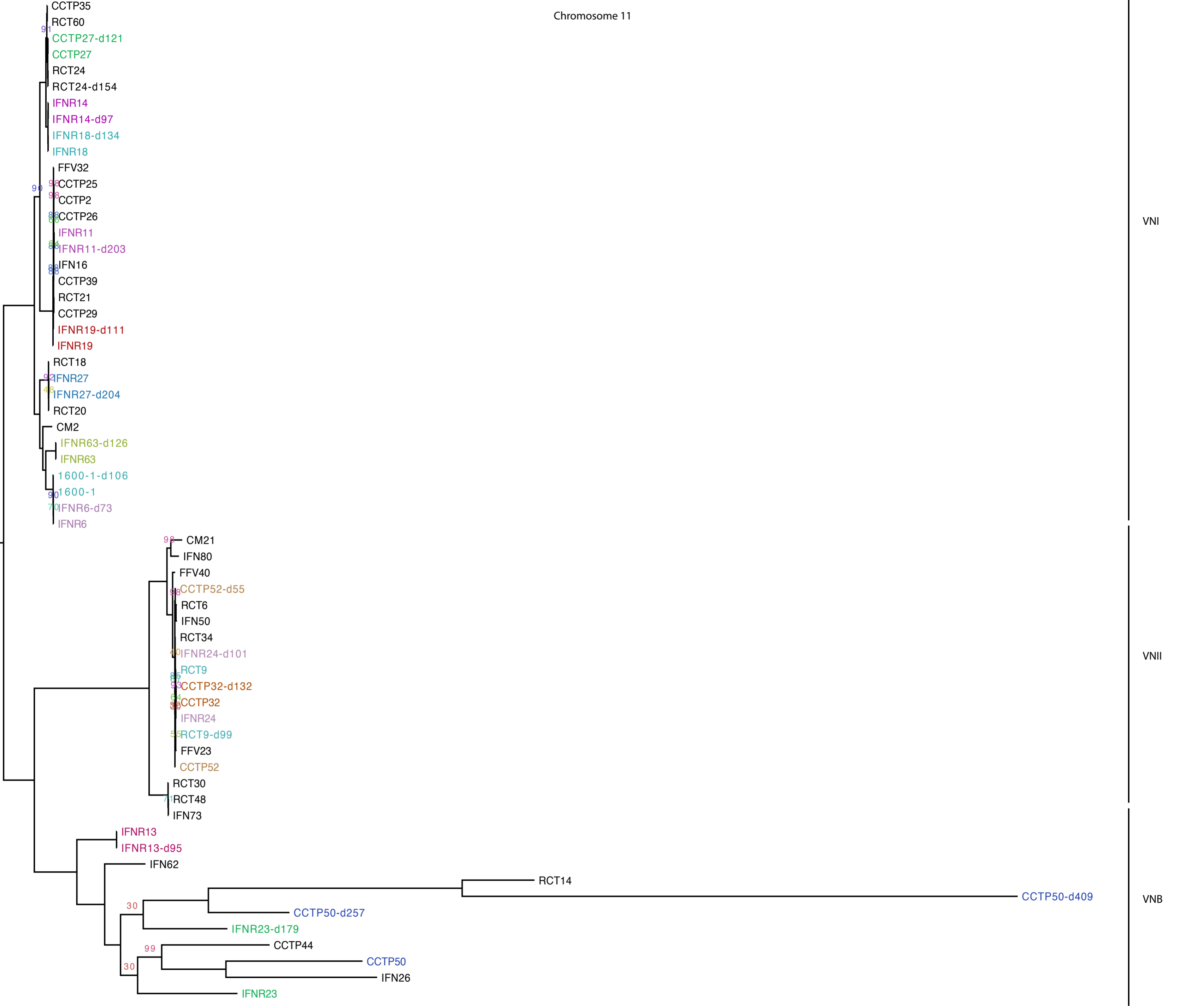

24000 SNPs

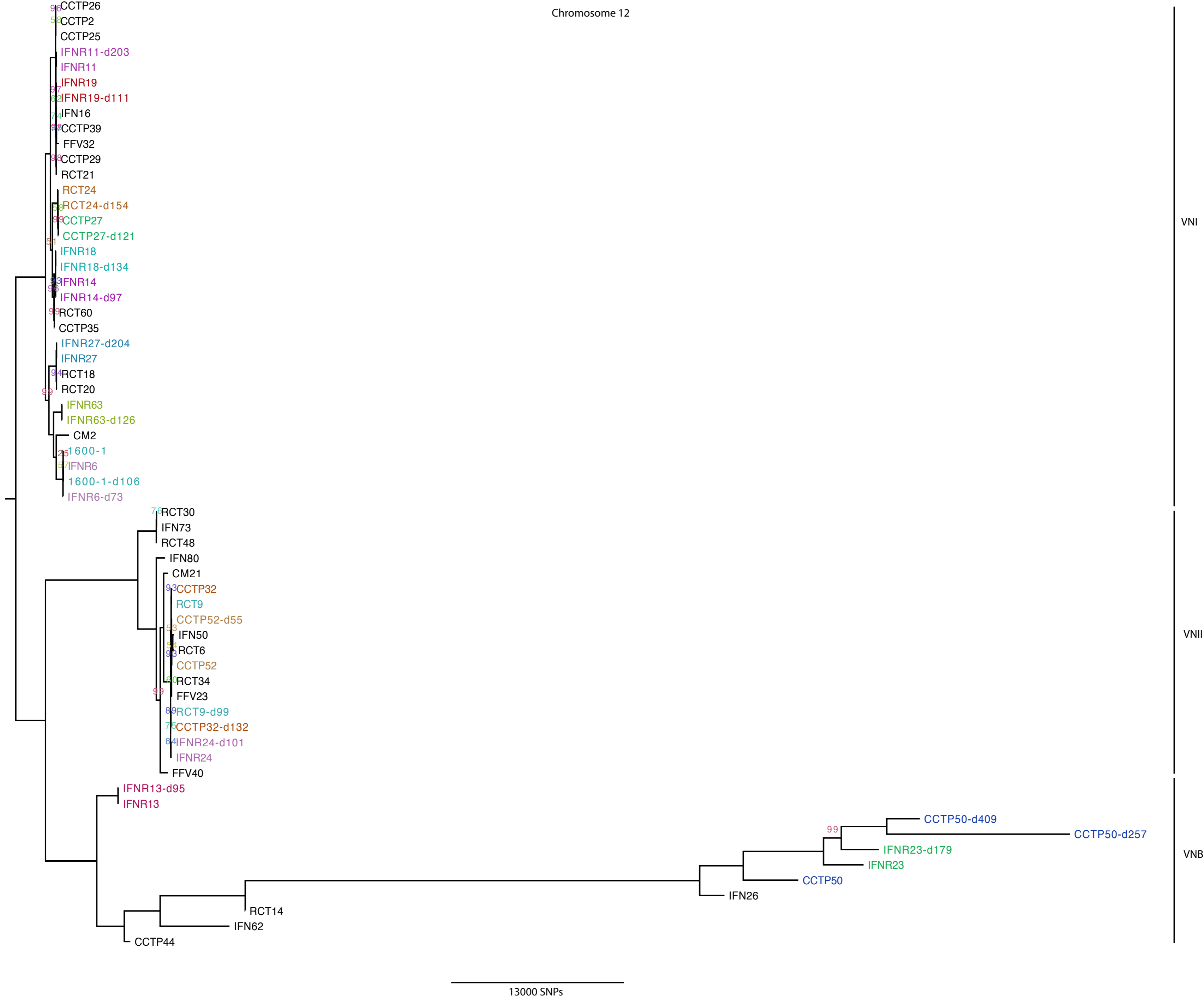

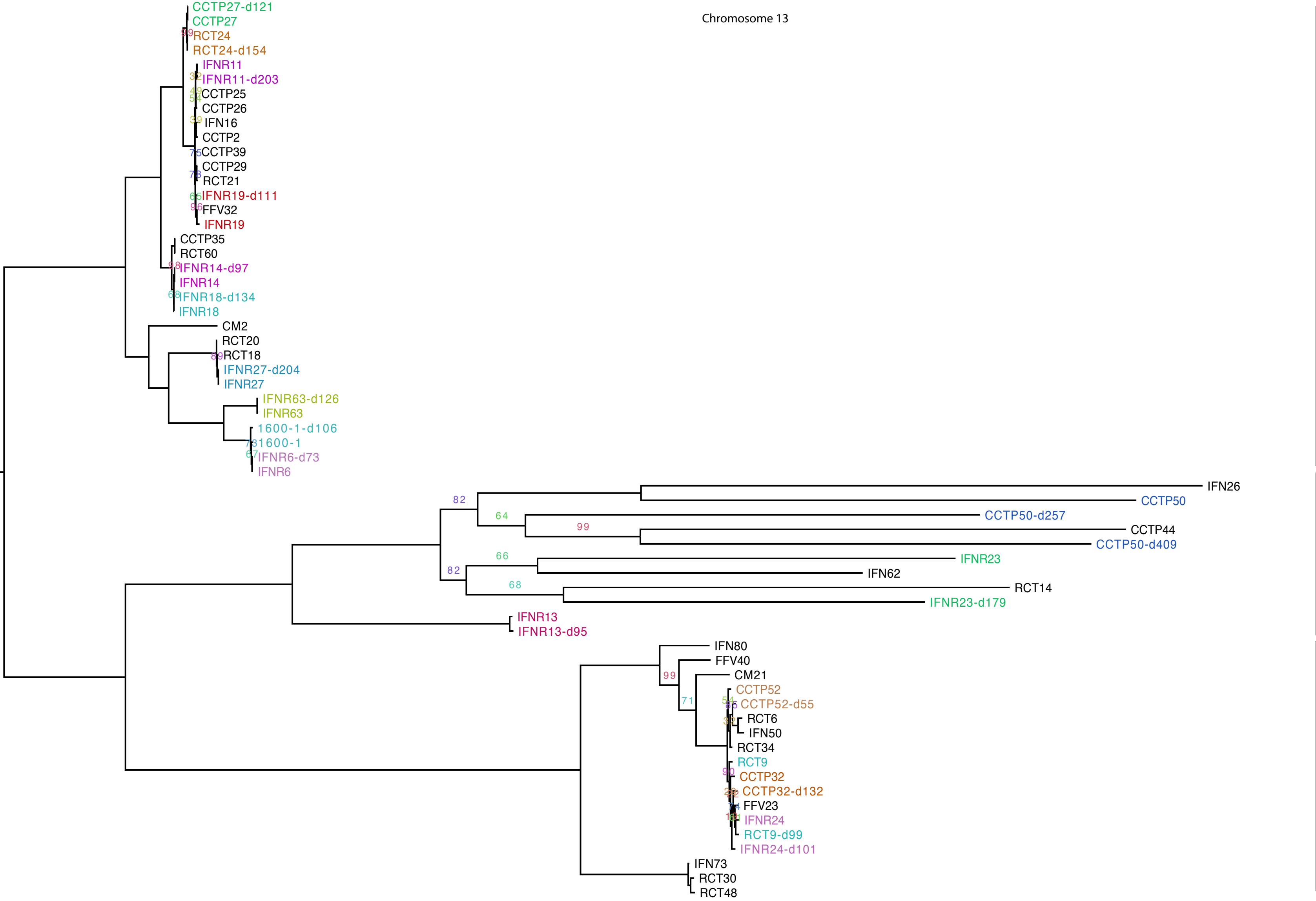

VNI

VNB

VNII

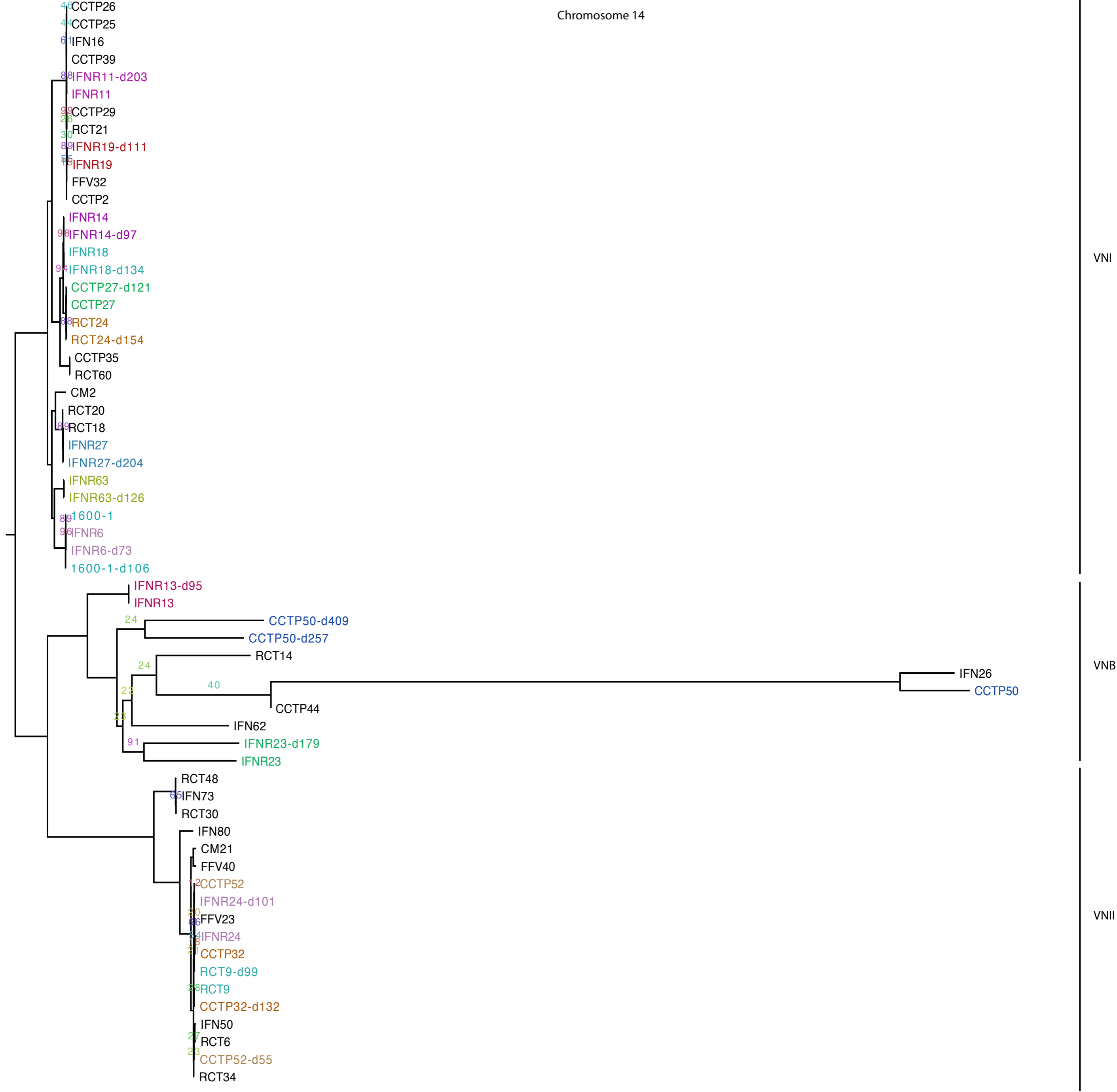

Supplement: Supplementary file 1 [file 1165FigureS1.pdf]
