# Supplementary material for: A Population Genomics Approach to Assessing the Genetic Basis of Within-Host Microevolution Underlying Recurrent Cryptococcal Meningitis Infection
Source: G3 (Bethesda). 2017 Feb 10;7(4):1165–76. doi: 10.1534/g3.116.037499 (PMC5386865; doi:10.1534/g3.116.037499)
Supplement: Supplementary file 2 [file 1165FigureS2.pdf]

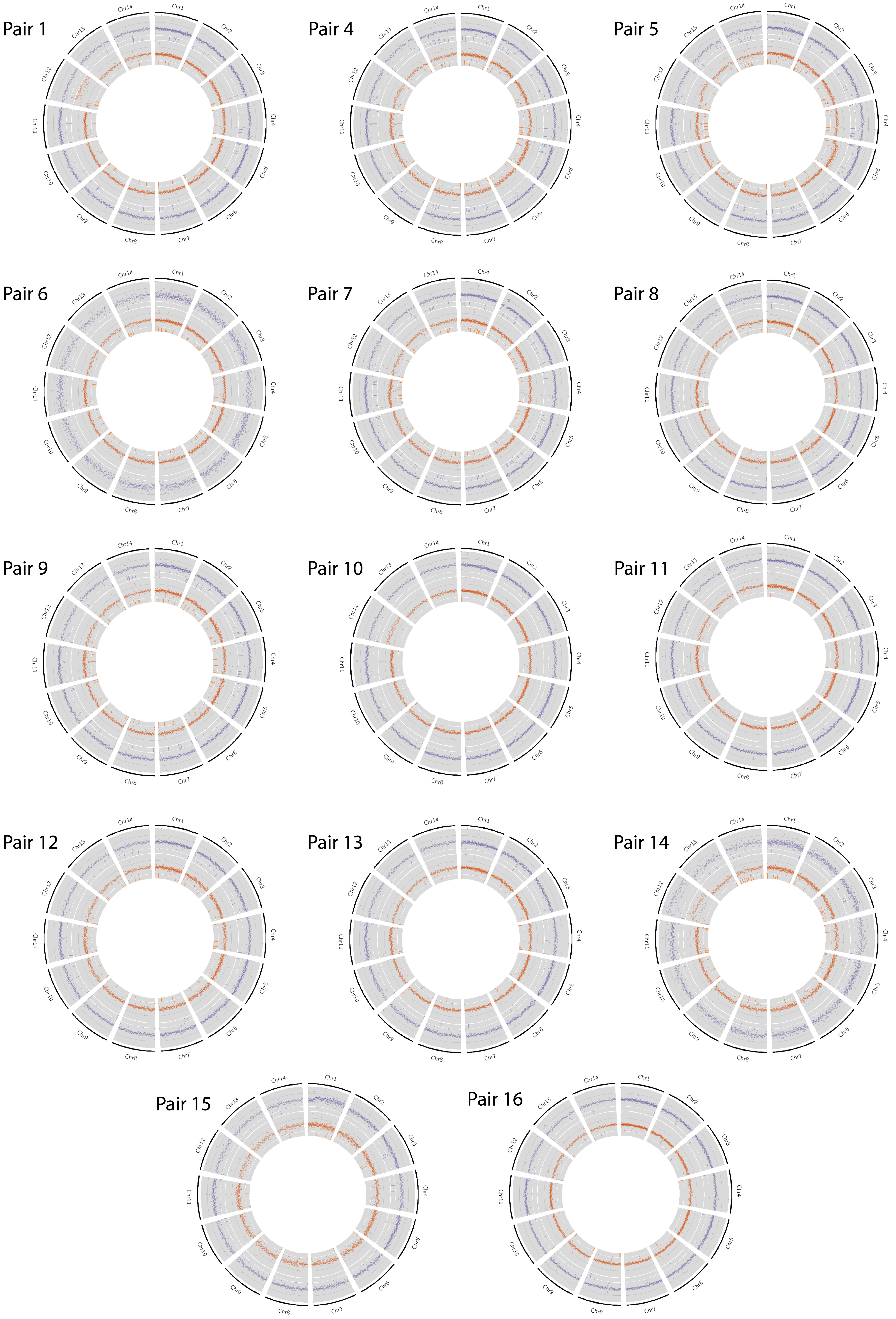
Pair 1
Pair 4
Pair 5
Pair 6
Pair 7
Pair 8
Pair 9
Pair 10
Pair 11
Pair 12
Pair 13
Pair 14
Pair 15
Pair 16
