# Supplementary material for: A Population Genomics Approach to Assessing the Genetic Basis of Within-Host Microevolution Underlying Recurrent Cryptococcal Meningitis Infection
Source: G3 (Bethesda). 2017 Feb 10;7(4):1165–76. doi: 10.1534/g3.116.037499 (PMC5386865; doi:10.1534/g3.116.037499)
Supplement: Supplementary file 3 [file 1165FileS1.docx]

Supplementary Material

Supplementary Table 1:

| Isolate ID | MLST colony 1 | MLST colony 2 | MLST colony 3 |
| --- | --- | --- | --- |
| CCTP27 | 234 | 1/63/212 | 1 |
| CCTP27-d121 | * | 1/63/212 | 1 |
| CCTP32 | 40 | 40/100/207/244 | 40 |
| CCTP32-d132 | * | 40/100/207/244 | 40 |
| CCTP50 | ** | ** | ** |
| CCTP50-d257 | * | ** | ** |
| CCTP50-d409 | * | ** | ** |
| CCTP52 | 40 | 40/100/207/244 | 40 |
| CCTP52-d55 | * | 40/100/207/244 | 40 |
| RCT9 | 40 | 40/100/207/244 | 40 |
| RCT9-d99 | * | 40/100/207/244 | 40 |
| RCT24 | 63 | 1/63/212 | 1 |
| RCT24-d154 | * | 1/63/212 | 1 |
| 1600-1 | * | * | 100/196 |
| 1600-1-d106 | * | * | 100/196 |
| IFNR63 | 32 | * | 32 |
| IFNR63-d126 | * | * | 32 |
| IFNR24 | 40 | * | 40 |
| IFNR24-d101 | * | * | 40 |
| IFNR18 | 23 | * | 23 |
| IFNR18-d134 | * | * | 23 |
| IFNR14 | 23 | * | 23 |
| IFNR14-d97 | * | * | 23 |
| IFNR13 | 247 | * | 247 |
| IFNR13-d95 | * | * | 247 |
| IFNR6 | 100 | * | 100 |
| IFNR6-d73 | * | * | 100 |
| IFNR19 | 71 | * | 71 |
| IFNR19-d111 | * | * | 71 |
| IFNR11 | 71 | * | 71 |
| IFNR11-d203 | * | * | 71 |
| IFNR27 | 5 | * | 5 |
| IFNR27-d204 | * | * | 5 |
| IFNR23 | ** | * | ** |
| IFNR23-d179 | ** | * | ** |

* Not tested

** Could not be determined (allele types could not be generated)

Supplementary Table 2:

| Isolate ID | SYN | NSY | Intron | Intergenic | p5UTR | P3UTR | NON | Total |
| --- | --- | --- | --- | --- | --- | --- | --- | --- |
| CCTP32 | 15 | 6 | 12 | 18 | 4 | 3 | 0 | 58 |
| CCTP32-d132 | 15 | 6 | 12 | 18 | 4 | 3 | 0 | 58 |
| CCTP50 | 14 | 3 | 13 | 18 | 2 | 3 | 0 | 53 |
| CCTP50-d257 | 14 | 3 | 13 | 18 | 2 | 3 | 0 | 53 |
| CCTP50-d409 | 14 | 3 | 13 | 18 | 2 | 3 | 0 | 53 |
| CCTP52 | 15 | 6 | 12 | 18 | 4 | 3 | 0 | 58 |
| CCTP52-d55 | 15 | 6 | 12 | 18 | 4 | 3 | 0 | 58 |
| RCT9 | 15 | 6 | 12 | 18 | 4 | 3 | 0 | 58 |
| RCT9-d99 | 15 | 6 | 12 | 18 | 4 | 3 | 0 | 58 |
| 1600-1 | 2 | 0 | 0 | 1 | 0 | 1 | 0 | 4 |
| 1600-1-d106 | 2 | 0 | 0 | 1 | 0 | 1 | 0 | 4 |
| IFNR63 | 2 | 2 | 1 | 2 | 0 | 0 | 0 | 7 |
| IFNR63-d126 | 2 | 2 | 1 | 2 | 0 | 0 | 0 | 7 |
| IFNR24 | 15 | 6 | 12 | 18 | 4 | 3 | 0 | 58 |
| IFNR24-d101 | 15 | 6 | 12 | 18 | 4 | 3 | 0 | 58 |
| IFNR14 | 0 | 0 | 1 | 0 | 0 | 0 | 0 | 1 |
| IFNR14-d97 | 0 | 0 | 1 | 0 | 0 | 0 | 0 | 1 |
| IFNR13 | 14 | 3 | 14 | 22 | 1 | 3 | 0 | 57 |
| IFNR13-d95 | 14 | 3 | 14 | 22 | 1 | 3 | 0 | 57 |
| IFNR6 | 2 | 0 | 0 | 1 | 0 | 1 | 0 | 4 |
| IFNR6-d73 | 2 | 0 | 0 | 1 | 0 | 1 | 0 | 4 |
| IFNR19 | 1 | 0 | 1 | 0 | 0 | 1 | 0 | 3 |
| IFNR19-d111 | 1 | 0 | 1 | 0 | 0 | 1 | 0 | 3 |
| IFNR11 | 1 | 0 | 1 | 0 | 0 | 1 | 0 | 3 |
| IFNR11-d203 | 1 | 0 | 1 | 0 | 0 | 1 | 0 | 3 |
| IFNR27 | 8 | 2 | 5 | 3 | 2 | 0 | 0 | 20 |
| IFNR27-d204 | 8 | 2 | 5 | 3 | 2 | 0 | 0 | 20 |
| IFNR23 | 137 | 19 | 110 | 18 | 4 | 3 | 2 | 293 |
| IFNR23-d179 | 138 | 19 | 107 | 18 | 5 | 4 | 2 | 293 |

Supplementary Table 3:

|  | Chromosome | | | | | | | | | | | | | |
| --- | --- | --- | --- | --- | --- | --- | --- | --- | --- | --- | --- | --- | --- | --- |
| Isolate | 1 | 2 | 3 | 4 | 5 | 6 | 7 | 8 | 9 | 10 | 11 | 12 | 13 | 14 |
| CCTP50 | 95 | 12683 | 6950 | 11566 | 1005 | 31 | 2705 | 14627 | 10980 | 9262 | 7031 | 7044 | 2840 | 10007 |
| CCTP50-d257 | 5 | 12769 | 6836 | 4027 | 8 | 2810 | 14367 | 5332 | 4534 | 3641 | 6125 | 8672 | 2465 | 3517 |
| CCTP50-d409 | 3 | 16362 | 16772 | 11553 | 36 | 3 | 14099 | 5401 | 13263 | 3730 | 16371 | 8284 | 2661 | 3830 |

Supplementary Table 4:

| Pair | Isolate ID | # Non-synonymous SNPs | # Synonymous SNPs |
| --- | --- | --- | --- |
| 1 | CCTP27 | 2927 | 3398 |
| 1 | CCTP27-d121 | 2937 | 3401 |
| 2 | CCTP32 | 54218 | 84080 |
| 2 | CCTP32-d132 | 54227 | 84080 |
| 3 | CCTP50 | 108662 | 216459 |
| 3 | CCTP50-d257 | 86611 | 174873 |
| 3 | CCTP50-d409 | 124501 | 253285 |
| 4 | CCTP52 | 54193 | 84074 |
| 4 | CCTP52-d55 | 54196 | 84075 |
| 5 | RCT9 | 54220 | 84056 |
| 5 | RCT9-d99 | 54225 | 84055 |
| 6 | RCT24 | 2854 | 3301 |
| 6 | RCT24-d154 | 2935 | 3369 |
| 7 | 1600-1 | 9867 | 12800 |
| 7 | 1600-1-d106 | 9878 | 12788 |
| 8 | IFNR63 | 9626 | 12453 |
| 8 | IFNR63-d126 | 9623 | 12455 |
| 9 | IFNR24 | 54242 | 84024 |
| 9 | IFNR24-d101 | 54196 | 84029 |
| 10 | IFNR18 | 2768 | 3326 |
| 10 | IFNR18-d134 | 2775 | 3331 |
| 11 | IFNR14 | 2773 | 3344 |
| 11 | IFNR14-d97 | 2769 | 3342 |
| 12 | IFNR13 | 38835 | 65817 |
| 12 | IFNR13-d95 | 38836 | 65823 |
| 13 | IFNR6 | 9869 | 12817 |
| 13 | IFNR6-d73 | 9866 | 12802 |
| 14 | IFNR19 | 6306 | 8016 |
| 14 | IFNR19-d111 | 6368 | 8039 |
| 15 | IFNR11 | 6371 | 8037 |
| 15 | IFNR11-d203 | 6367 | 8038 |
| 16 | IFNR27 | 9382 | 12323 |
| 16 | IFNR27-d204 | 9382 | 12305 |
| 17 | IFNR23 | 94116 | 190717 |
| 17 | IFNR23-d179 | 105639 | 209910 |

Supplementary Table 5:

| Pair # | Isolate ID | # reads aligned (millions) | Depth of coverage (x) | % reference genome covered^a^ | # filtered SNPs |
| --- | --- | --- | --- | --- | --- |
| 1 | CCTP27 | 16.3 | 83 | 99.6 | 13587 |
| 1 | CCTP27-d121 | 19.2 | 98 | 99.6 | 13600 |
| 2 | CCTP32 | 20.9 | 105 | 97.6 | 290637 |
| 2 | CCTP32-d132 | 23.4 | 117 | 97.7 | 290548 |
| 3 | CCTP50 | 14.7 | 68 | 99.2 | 559992 |
| 3 | CCTP50-d257 | 18.8 | 88 | 96.9 | 461182 |
| 3 | CCTP50-d409 | 18.9 | 88 | 97.5 | 646454 |
| 4 | CCTP52 | 31.3 | 155 | 97.7 | 290743 |
| 4 | CCTP52-d55 | 17.2 | 84 | 97.6 | 290670 |
| 5 | RCT9 | 19.3 | 97 | 97.6 | 290628 |
| 5 | RCT9-d99 | 20.3 | 102 | 97.6 | 290447 |
| 6 | RCT24 | 3.1 | 15 | 99.5 | 13255 |
| 6 | RCT24-d154 | 20.2 | 103 | 99.6 | 13567 |
| 7 | 1600-1 | 21.8 | 111 | 99.4 | 48355 |
| 7 | 1600-1-d106 | 16.6 | 84 | 99.4 | 48370 |
| 8 | IFNR63 | 33.9 | 172 | 99.6 | 47177 |
| 8 | IFNR63-d128 | 22.2 | 113 | 99.5 | 47179 |
| 9 | IFNR24 | 19.3 | 97 | 97.6 | 290491 |
| 9 | IFNR24-d101 | 19.1 | 96 | 97.6 | 290567 |
| 10 | IFNR18 | 20.1 | 102 | 99.7 | 12791 |
| 10 | IFNR18-d134 | 19.1 | 96 | 99.6 | 12804 |
| 11 | IFNR14 | 23.4 | 119 | 99.7 | 12842 |
| 11 | IFNR14-d97 | 20.7 | 105 | 99.7 | 12831 |
| 12 | IFNR13 | 18.4 | 92 | 98.7 | 222609 |
| 12 | IFNR13-d95 | 17.2 | 86 | 98.8 | 222751 |
| 13 | IFNR6 | 17.7 | 89 | 99.4 | 48458 |
| 13 | IFNR6-d73 | 20.0 | 101 | 99.4 | 48356 |
| 14 | IFNR19 | 7.6 | 36 | 99.4 | 29248 |
| 14 | IFNR19-d111 | 26.3 | 126 | 99.8 | 29986 |
| 15 | IFNR11 | 30.0 | 144 | 99.7 | 29990 |
| 15 | IFNR11-d203 | 25.2 | 121 | 99.7 | 29964 |
| 16 | IFNR27 | 27.8 | 141 | 99.6 | 45762 |
| 16 | IFNR27-d204 | 33.7 | 171 | 99.5 | 45778 |
| 17 | IFNR23 | 25.1 | 116 | 98.0 | 494004 |
| 17 | IFNR23-d179 | 26.7 | 124 | 97.6 | 549927 |

^a^ The Cng reference genome H99 was used to align reads and calculate the corresponding depth of coverage and percentage of the reference covered by reads.

Supplementary Table 6:

| Pair # | Isolate ID | Homozygous SNPs | Heterozygous SNPs |
| --- | --- | --- | --- |
| 3 | CCTP50 | 153953 | 1241341 |
| 3 | CCTP50-d257 | 290685 | 965881 |
| 3 | CCTP50-d409 | 257480 | 890871 |
| 4 | CCTP52 | 329681 | 11363 |
| 4 | CCTP52-d55 | 328207 | 9935 |
| 5 | RCT9 | 329047 | 10052 |
| 5 | RCT9-d99 | 328685 | 10109 |
| 17 | IFNR23 | 220234 | 1186119 |
| 17 | IFNR23-d179 | 265317 | 1147900 |
